# Detection of thermogenesis in rodents in response to anti-obesity drugs and genetic modification

**DOI:** 10.3389/fphys.2013.00064

**Published:** 2013-04-08

**Authors:** Jonathan R. S. Arch, Paul Trayhurn

**Affiliations:** Clore Laboratory, University of BuckinghamBuckingham, UK

**Keywords:** thermogenesis, energy expenditure, leanness, anti-obesity drug, genetically modified mouse, sympathomimetic, brown adipose tissue, leptin

## Abstract

Many compounds and genetic manipulations are claimed to confer resistance to obesity in rodents by raising energy expenditure. Examples taken from recent and older literature, demonstrate that such claims are often based on measurements of energy expenditure after body composition has changed, and depend on comparisons of energy expenditure divided by body weight. This is misleading because white adipose tissue has less influence than lean tissue on energy expenditure. Application of this approach to human data would suggest that human obesity is usually due to a low metabolic rate, which is not an accepted view. Increased energy expenditure per animal is a surer way of demonstrating thermogenesis, but even then it is important to know whether this is due to altered body composition (repartitioning), or increased locomotor activity rather than thermogenesis *per se*. Regression analysis offers other approaches. The thermogenic response to some compounds has a rapid onset and so cannot be due to altered body composition. These compounds usually mimic or activate the sympathetic nervous system. Thermogenesis occurs in, but may not be confined to, brown adipose tissue. It should not be assumed that weight loss in response to these treatments is due to thermogenesis unless there is a sustained increase in 24-h energy expenditure. Thyroid hormones and fibroblast growth factor 21 also raise energy expenditure before they affect body composition. Some treatments and genetic modifications alter the diurnal rhythm of energy expenditure. It is important to establish whether this is due to altered locomotor activity or efficiency of locomotion. There are no good examples of compounds that do not affect short-term energy expenditure but have a delayed effect. How and under what conditions a genetic modification or compound increases energy expenditure influences the decision on whether to seek drugs for the target or take a candidate drug into clinical studies.

## Introduction

Despite the continuing rise in the worldwide prevalence of obesity and the vast sales that a safe and effective drug for this disorder might achieve, many pharmaceutical companies have been disinclined to invest in research and development in this field in recent years because the task seemed near impossible. Following the withdrawal of fenfluramine and dexfenfluramine in 1997, primarily because they caused heart valve disease, sibutramine, and rimonabant offered a glimmer of hope, but either they never reached the US or European markets, or they were soon withdrawn owing to adverse cardiovascular or CNS side-effects. Only the pancreatic lipase inhibitor orlistat remains for long term pharmacotherapy, and its efficacy (about 3 kg weight loss compared to placebo when used at its highest approved dose) is limited (Rucker et al., 2007).

Some optimism has returned recently following the approval by the US Food and Drug Administration (FDA) of the combination of the anti-epileptic drug topiramate in combination with the “anorectic” drug phentermine under the brand name Qsymia. The European Medicines Agency has rejected Qsymia, however. Soon after its approval of Qsymia, the FDA also approved the selective 5HT_2C_ receptor agonist lorcaserin (Wong et al., 2012). Looking to the future, approval of the glucagon-like peptide-1 analogue liraglutide may be helped by it being already marketed for the treatment of type 2 diabetes, albeit at a lower dose than that being evaluated in Phase III clinical trials for obesity.

Apart from orlistat, the primary mechanism of which is to reduce energy absorption rather than intake, these drugs have all been perceived as anorectic agents. Is this entirely true? Studies in rodents suggest that the anti-obesity effects of many “anorectic” drugs are partly, or even entirely, due to increased energy expenditure (“thermogenesis”) (Arch, 1981; Day and Bailey, 1998; Picard et al., 2000; Herling et al., 2008). There is nothing new in the concept of thermogenic drugs: two of the earliest drugs for obesity of the scientific era—dinitrophenol and thyroid hormones—stimulate thermogenesis (Clapham and Arch, 2007).

It is important to understand how evidence that compounds are thermogenic in rodents has been obtained and whether this evidence translates to humans, because the question “Is it anorectic or thermogenic?” will continue to be asked of new drugs, drug candidates and drug targets. This question is especially pertinent because interest in thermogenic drugs and drug targets has been rekindled by new evidence that brown adipose tissue can be active in adult humans and the discovery of new targets for drugs that might augment and activate brown adipose tissue (Fruhbeck et al., 2009; Wu et al., 2011; Bostrom et al., 2012; Fournier et al., 2012; Ye et al., 2012). The role of brown adipose tissue is to oxidise fat without coupling the energy released to the synthesis of ATP. The purpose of this uncoupling is either to produce heat (in which case fat loss can be seen as a by-product) or to regulate body fat stores (with thermogenesis as a by-product). Thus, drugs that target brown adipose tissue should be thermogenic.

The aim of this article is to discuss how thermogenesis in response to treatment with a drug can be detected. In addition, since targets for thermogenic drugs are often validated by investigating the phenotype of genetically modified mice, it considers how to ascertain whether leanness in a genetically modified animal is associated with increased energy expenditure. Examples of various approaches are taken from both old and recent literature. We argue that some of the claims are unjustified. The issue of how to compare the energy expenditure of lean and obese rodents has been discussed extensively elsewhere (Arch et al., 2006; Butler and Kozak, 2010; Kaiyala et al., 2010; Cannon and Nedergaard, 2011; Even and Nadkarni, 2012; Tschop et al., 2012; Speakman, 2013), so we shall not repeat all the arguments, but raise points that others may not have addressed. We are among those who object strongly to the expression of energy expenditure relative to body weight or body weight^0.75^, for the purpose of comparing lean and obese rodents. This is standard practice in most journals and apparently accepted without question by most referees, but it is often misleading or simply wrong—particularly and paradoxically since intake is almost invariably expressed on a “per animal” basis. Thus, a continuing theme is the dismissal of claims based on this practice. The claims that we have selected to dismiss are merely examples, often taken from recent literature. A number of other examples have been described in previous publications (Butler and Kozak, 2010; Arch, 2011), but even taking the articles together, these are only the tip of the iceberg. The translation of studies in rodents to humans is also briefly considered.

## Acute stimulation of energy expenditure

### Rapid onset response to compounds

#### Sympathomimetic agents

It is easiest to make the case for a compound being thermogenic if energy expenditure rises rapidly, ideally within minutes after its administration. This has two advantages over other types of evidence: energy expenditure prior to administration of the compound provides a baseline control, and interpretation of data is not complicated by changes in body weight or composition, because these are no different for the compound- and vehicle-treated animals.

Classic examples of such evidence are the rises in energy expenditure within an hour (or minutes if given intravenously) following administration of noradrenaline, and sympathomimetic compounds such as phentermine, ephedrine and β_3_-adrenoceptor agonists (Arch, 1981; Arch et al., 1982, 1984; Wilson et al., 1984; Holloway et al., 1991; Granneman et al., 2003; Kong et al., 2004). The rapid increases in energy expenditure and brown adipose tissue temperature elicited by caffeine (Arch et al., 1987; Yoshioka et al., 1990), theophylline (Strubelt and Siegers, 1969), green tea (Dulloo et al., 2000; Choo, 2003) and nicotine (Wellman et al., 1986; Collins et al., 1996a) are also probably mainly a consequence of their raising sympathetic activity, though other mechanisms—including increased locomotor activity in response to caffeine—may contribute (Arch et al., 1987). Sibutramine similarly elicits a slightly delayed, increase in energy expenditure in rodents as a consequence of it activating the sympathetic nervous system. It took about an hour for this effect to become statistically significant following intraperitoneal administration to rats, presumably because sibutramine must increase synaptic serotonin and noradrenaline concentrations in the hypothalamus before the sympathetic nervous system is activated. The simultaneous injection of the noradrenaline re-uptake inhibitor nisoxetine and the serotonin re-uptake inhibitor fluoxetine stimulated energy expenditure with a similar delay (Connoley et al., 1999). A more recent example is that intracerebroventricular administration of bone morphogenetic protein 8B elicited an increase in sympathetic activity in brown adipose tissue and in core temperature within an hour, but energy expenditure was not measured directly (Whittle et al., 2012). By contrast, zinc-α_2_-glycoprotein (ZAG), despite being claimed to be a β_3_-adrenoceptor agonist (Russell et al., 2002; Russell and Tisdale, 2012a), did not elicit a rapid rise in energy expenditure in our hands (Wargent et al., 2013), and has not been reported to do so by others. Nevertheless, it may raise energy expenditure over a period of days and reduce body weight and fat independently of any effect on food intake (Russell and Tisdale, 2011a,b).

A possible reason why sympathomimetic agents have such a marked thermogenic effect is that they stimulate the mobilization of fatty acids as well as their combustion. Thus, a β_3_-adrenoceptor agonist stimulated thermogenesis in mice that expressed the β_3_-adrenoceptor in brown and white adipose tissue (and no other tissue), but it had little effect when the β_3_-adrenoceptor was expressed in brown adipose tissue only—the brown adipocytes seemed to be starved of fuel to burn (Grujic et al., 1997). Similarly, antilipolytic agents, such as nicotinic acid, reduce thermogenesis in response to catecholamines and β-adrenoceptor agonists (Kennedy and Ellis, 1969; Lafrance et al., 1979; Schiffelers et al., 1998), and the thermogenic effects of β_3_-adrenoceptor agonists are less prolonged in lean or lipoatrophic mice than in obese mice (Arch and Ainsworth, 1983a; Gavrilova et al., 2000).

It is reasonable to consider whether any compound that elicits a rapid increase in energy expenditure does so by activating the sympathetic nervous system. One such compound is the cannabinoid 1-receptor (CB1-R) antagonist rimonabant, which elicits a rapid increase in energy expenditure in rats (Herling et al., 2008; Kunz et al., 2008), though this has not been demonstrated in humans. Antagonism of the CB1-R promotes noradrenaline release at peripheral sympathetic nerves (Marsicano and Lutz, 2006; Mnich et al., 2010) and activates sympathetic activity via a central mechanism (Verty et al., 2009). It is therefore possible that the acute thermogenic effect of rimonabant in rodents is mediated by the sympathetic nervous system. Other examples of rapid thermogenic responses that are probably mediated by raised sympathetic activity are those to a catalytic antibody that hydrolyzed the octanoyl moiety of ghrelin (Mayorov et al., 2008; Arch, 2011), to amylin (Osaka et al., 2008) and to thyrotrophin-releasing hormone (Schuhler et al., 2007). In the case of thyrotrophin-releasing hormone, evidence for the involvement of the sympathetic nervous system is that its intracerebroventricular infusion increased noradrenaline turnover in brown adipose tissue, and thermogenesis was suppressed by sympathetic denervation of brown adipose tissue (Shintani et al., 2005).

The thermogenic effects of β_3_-adrenoceptor agonists and amylin, and the anti-obesity effect of ZAG are reduced or prevented by the β-adrenoceptor antagonist propranolol (Arch and Ainsworth, 1983a; Arch et al., 1991; Osaka et al., 2008; Russell and Tisdale, 2012a,b). We have suggested that the anti-obesity effect of ZAG may be due to central activation of the sympathetic nervous system, rather than direct activation of the β_3_-adrenoceptor and this is why its anti-obesity effect is blocked by propranolol (Wargent et al., 2013). Propranolol also blocked thermogenesis in response to the centrally acting sympathomimetic agents caffeine and theophylline (Strubelt and Siegers, 1969). Doses of β-adrenoceptor antagonists have to be high to block the rodent β_3_-adrenoceptor and there is therefore a risk that they might elicit non-β-adrenoceptor-mediated effects.

Alternative approaches to investigating the role of the sympathetic nervous system can be illustrated by considering studies on leptin. Intracerebroventricular administration of leptin increases energy expenditure in *ob/ob* mice within 3 h (Mistry et al., 1997). In rats, intracerebroventricular injection of leptin increased body temperature after an hour; peripheral administration took 3 h (Luheshi et al., 1999). Subcutaneous administration has been shown to increase energy expenditure within 12 h in suckling rats (Stehling et al., 1996) and 2 days when infused by minipump in normal mice (Asensio et al., 2008). Leptin increases noradrenaline turnover in brown and white adipose tissue (Collins et al., 1996b) and electrical activity in sympathetic nerves (Dunbar et al., 1997; Hausberg et al., 2002). None of this shows whether increased sympathetic activity is responsible for any of the thermogenic activity of leptin, however. Better evidence is that the thermogenic effect on days 2–6 of subcutaneously infused leptin was reduced by about 50% in mice that lack all three β-adrenoceptors (betaless mice) (Asensio et al., 2008). Work in *Lep*^°*b*^/*Lep*^°*b*^ mice that also lack UCP-1 suggests that elevation of plasma tri-iodothyronine by leptin and activation of sarcoendoplasmic reticulum Ca^2+^ ATPase may contribute to some of the remaining thermogenic effect of leptin (Ukropec et al., 2006b). Studies on the role of the sympathetic nervous system in the anti-obesity effect of leptin are described in section Detection of Non-Acute Thermogenesis.

Despite these inconclusive results with leptin, it is advisable to investigate whether a prospective thermogenic anti-obesity compound is as effective in sympathectomised, betaless, or individual β-adrenoceptor knockout mice as it is in normal mice. This information may help in the design of clinical studies (see section Translation of Rodent Findings to Humans). It might also be useful to evaluate compounds in mice that lack brown adipose tissue (Lowell et al., 1993), though it should not be assumed that all sympathetically-mediated thermogenesis in rodents is in brown adipose tissue (Thurlby and Ellis, 1986). Even those who argue that all *adaptive* thermogenesis is in brown adipose tissue find that much of the thermogenic response to noradrenaline in mice that are fed on a normal diet and housed at thermoneutrality remains in mice that lack uncoupling protein-1 (UCP-1), thereby lacking the defining protein of brown adipose tissue (Feldmann et al., 2009). They argue that the UCP-1-independent thermogenic effect of noradrenaline is a pharmacological, non-adaptive effect that takes place in tissues other than brown adipose tissue, rather than a physiological effect (Cannon and Nedergaard, 2011). These arguments are consistent with adaptive thermogenesis being exclusive to brown adipose tissue (Cannon and Nedergaard, 2011) and with reports that thermogenic doses of the β-adrenoceptor agonist isoprenaline and the sympathomimetic drug ephedrine failed to activate brown adipose tissue in humans, although cold exposure did (Cypess et al., 2012; Vosselman et al., 2012). A non-physiological mechanism of action is less attractive in a drug than a physiological mechanism. On the other hand, it seems unlikely that thermogenesis in tissues other than brown adipose tissue is entirely non-physiological, because exposure to cold for 4 days increased triacylglycerol/fatty acid substrate cycling in white as well as brown adipose tissue (Brooks et al., 1983). Other substrate cycles—sometimes called “futile” cycles—may also be stimulated in the cold, even though their primary function may not be thermogenesis. UCP-1 knockout mice are able to increase their response to cold exposure when acclimated to cold, and there is evidence that this is partly due to increased oxidative capacity and ATP utilization in white adipose tissue (Meyer et al., 2010; Ukropec et al., 2006a). Cold exposure (for 2.5 days) also increased fructose-6-phosphate/fructose-1,6-bisphosphate cycling in skeletal muscle but this might have been a consequence of shivering *in vivo*, even though cycling was measured *in vitro* (Challis et al., 1985). On the other hand, when A/J mice were fed on a high fat diet, soleus muscles taken from them had an increased oxygen consumption, which cannot be attributed to shivering (Kus et al., 2008). Moreover, mice in which sarcolipin is absent from skeletal muscle were less able than wild-type mice to defend their body temperature in the cold, even when the mice are treated with curare to prevent shivering, suggesting that futile pumping of Ca^2+^ may play a role in thermogenesis (Bal et al., 2012). Finally, it is worth noting that by contrast with the recent reports that isoprenaline and ephedrine stimulated thermogenesis without activating brown fat in humans (Cypess et al., 2012; Vosselman et al., 2012), noradrenaline stimulated thermogenesis in both brown adipose tissue and the hind limb (primarily skeletal muscle) in rodents (Thurlby and Ellis, 1986), suggesting that brown fat as well as other tissues is physiologically relevant in rodents.

#### Non-sympathomimetic mechanisms

Non-sympathetic mechanisms may also elicit rapid rises in energy expenditure. Sympathomimetic compounds stimulate thermogenesis primarily via β-adrenoceptors and Gα_s_. One would therefore expect that activation of any Gα_s_-coupled receptor in brown adipose tissue should rapidly activate energy expenditure. The bile acid receptor TGR5 is an example of such a receptor. Agonists of TGR5 increase the concentration of cyclic AMP in isolated brown adipocytes within 1 h (Watanabe et al., 2006). Surprisingly, however, a rapid rise in energy expenditure in response to administration of a TGR5 agonist has not been described. Instead, the chronic thermogenic activity of TGR5 agonists has been ascribed to increased expression of type 2 iodothyronine deiodinase (D2), which converts thyroxine to triiodothyronine, because the TGR5 agonist cholic acid did not increase diet-induced thermogenesis in mice that lack D2. This is not a strong argument because β_3_-adrenoceptor agonists also have little thermogenic activity in the absence of a functional thyroid system (Rubio et al., 1995; Golozoubova et al., 2004).

Activation of AMP-activated protein kinase (AMPK) in peripheral tissues promotes metabolic pathways that result in ATP production, whilst inhibiting those that require ATP utilization. Consistent with this role, nootkatone, a constituent of grapefruit that appears to activate kinases that are upstream of AMPK, elicited a rapid increase in energy expenditure in mice that was not associated with increased locomotor activity (Murase et al., 2010). Similar acute effects have not been described for directly acting AMPK activators, such as 5-aminoimidazole-4-carboxamide-1-β-D-ribofuranoside (AICAR) or the thienopyridone A769662; nor for metformin, which activates AMPK by raising the tissue AMP concentration (Hardie, 2008). However, both A769662 and metformin reduced the respiratory exchange ratio (RER) of rats for about 3 h, after which the ratio increased for about 3 h (Cool et al., 2006), suggesting depletion of fat due to its increased oxidation or decreased synthesis. Metformin may cause a slight reduction in body weight in humans (Golay, 2008), but this seems to be due to decreased energy intake rather than increased energy expenditure. As in rats, however, metformin causes a transient reduction in RER (Arch, 2011). By comparison with other activators of AMPK, the rapid response to nootkatone is so unusual that it would be logical to check whether it might be a consequence of sympathetic activation.

Thyroid hormones and possibly fibroblast growth factor 21 (FGF21) are examples of treatments that bridge the gap between sympathomimetic compounds, which elicit rapid increases in energy expenditure, and some of the compounds and genetic modifications that are described in section Detection of Non-Acute Thermogenesis. In both cases, increased energy expenditure has been dissociated from any discernible effect on body weight, so it is unlikely that the increase in energy expenditure was a consequence of a change in body composition.

Depending on the dose, the onset of the response to thyroid hormones has been reported as 18 h to more than 5 days (Myant and Witney, 1967; de Lange et al., 2001; Kong et al., 2004). A variety of mechanisms, involving both decreased efficiency of ATP production and increased ATP utilization, for example activation sarcoendoplasmic reticulum Ca^2+^ ATPase (Silva, 2006; Ukropec et al., 2006b), have been suggested to explain thyroid-hormone induced thermogenesis, but it should also be remembered that thyroid hormones increase thermogenic responsiveness to the sympathetic nervous system (Ribeiro et al., 2001, 2010), once again suggesting that the sympathetic nervous system may play a role in thermogenesis.

FGF21 is released from liver and from brown and white adipocytes (Muise et al., 2008). Increased energy expenditure per animal was detected 2 days after intraperitoneal administration of FGF21 to diet-induced mice (Xu et al., 2009). The thermogenic effect of exogenous FGF21 may be a consequence of activation of brown adipose tissue, the induction of genes involved in oxidative metabolism in liver and adipose tissue, and the conversion of white adipocytes to cells that have some of the characteristics of brown adipocytes (Xu et al., 2009; Hondares et al., 2010; Fisher et al., 2012). The development of FGF21 itself as a drug presents significant challenges but recently workers from Genentech have described monoclonal antibodies that activate the FGF receptor 1 and have an antidiabetic effect in mice and monkeys (Wu et al., 2011; Foltz et al., 2012).

Obviously, compounds may elicit rapid rises in energy expenditure by promoting motor activity. Speakman (2013) discusses methods for measuring resting energy expenditure (REE), which should allow such a mechanism to be excluded.

### Technical issues

The evidence that the compounds described above elicit a rapid rise in energy expenditure has mostly been obtained using open-circuit indirect calorimetry. This involves passing air through a respiratory chamber and comparing its oxygen concentration after leaving the chamber with that of air entering the chamber (or leaving a chamber that contains no animals). The difference in these oxygen concentrations, coupled with the rate flow of air exiting the chamber cannot be used to measure oxygen consumption accurately because the flow of air into the chamber equals the flow out only when the RER = 1 (carbohydrate oxidation). When the RER is 0.72 (fat oxidation), oxygen consumption is underestimated by 6% if the two flow rates are assumed to be equal, because flow of air and therefore the amount of oxygen entering the chamber is higher than assumed. Fortunately, for the same consumption of oxygen, fat provides 6% less energy that carbohydrate. Consequently, calculation of energy expenditure assuming that only carbohydrate is being oxidized provides a very accurate measure of energy expenditure, whatever the balance of fuels. In other words, thanks to a mathematical fluke, measurement of oxygen concentration alone gives a more accurate measure of energy expenditure than of oxygen consumption. If the carbon dioxide concentration of the air leaving the chamber is also measured, it is possible to determine both carbon dioxide production and oxygen consumption and therefore the RER (Arch et al., 2006).

Changes in energy expenditure in response to specific compounds may be more rapid than they appear to be in publications. First, it takes a short time for changes in tissue oxygen utilization and carbon dioxide production to be reflected in expired air and then for any of that air to reach the gas analyzers. More importantly, most authors do not correct their calculation of energy expenditure to take account of the time that it takes for a change in energy expenditure to be fully reflected in the composition of the gases in the respiratory chamber. They ignore the fact that the volume of oxygen consumed is not only the difference between that entering and leaving the chamber: the decrease or increase in the amount of oxygen in the chamber must also be taken into account. Similarly, carbon dioxide production is not just the difference between the amount leaving and entering the chamber. Assuming perfect mixing of the gases in the respiratory chamber, the half-life of the approach to the situation where the amount of oxygen in the chamber is constant after a step change in energy expenditure is 0.693 × chamber volume/flow rates. It is possible to calculate instantaneous energy expenditure by applying a correction derived from the rate of change of the difference between the oxygen concentration of air entering and leaving the chamber, but this can only be accurate if each chamber is monitored continuously (Arch et al., 2006; Speakman, 2013).

In our case, we keep animals in their home cages during the measurement of energy expenditure. If the chamber volume is 20 l and the flow rate is 0.5 l/min, the half-life of the approach to steady state is 28 min—far too long to compare energy expenditure with instantaneous measures of physical activity. Moreover, we do not monitor each chamber continuously. Others use smaller chamber volumes, so this is not so much of an issue. Nevertheless, it would be helpful if authors would describe both the chamber volume and the flow rate, and state whether their calculations of energy expenditure are corrected for the steady state issue.

### Surrogate markers of thermogenesis

Not everybody has access to indirect calorimetry. This raises the question of what measurements might be used as surrogate markers of thermogenesis. Nothing can substitute for calorimetry as a quantitative measure of thermogenesis, but an elevation of the temperature of the heat-producing tissue, ideally relative to core temperature (Wellman and Marmon, 1985; Yoshioka et al., 1990), is good indication that thermogenesis is taking place. Occasionally, brown adipose tissue temperature alone is measured (Ueta et al., 2012), but measurement of core temperature alone, being technically easier, is far more common (Malinowska and Schlicker, 1997; Connoley et al., 1999; Luheshi et al., 1999; Russell and Tisdale, 2012a). Measurement of core temperature does not make the assumption that brown adipose tissue is the site of thermogenesis, but it ignores the possibility that there is a centrally-mediated increase in the set point of body temperature, as occurs in fever. An increase in body temperature due to a centrally-mediated increase in the set point of body temperature might be achieved by means of decreased heat loss rather than increased energy expenditure. Decreased heat loss becomes more important at higher ambient temperatures because it is at these temperatures that skin blood flow and evaporative water loss come into play as cooling mechanisms (Gordon, 2012).

Some workers have combined measurement of core temperature with exposure of animals to cold (typically 4–10°C). This approach has helped identify targets for thermogenic drugs in both brown adipose tissue and muscle (Bal et al., 2012; Fournier et al., 2012). Another surrogate is increased sympathetic activity, especially in brown adipose tissue, which has already been mentioned as an effect of bone morphogenetic protein 8B (Whittle et al., 2012).

Increased expression of the UCP1 gene, which may be detectable at the protein level within a day (Klein et al., 2000) and much earlier at the mRNA level, has been used by many workers as evidence that brown adipose tissue thermogenesis has been activated. A note of caution, however: peroxisome proliferator-activated receptor-γ agonists increase the expression of UCP-1 in brown adipose tissue, but they do not increase thermogenesis because sympathetic activity is reduced (Festuccia et al., 2008). Increased expression of UCP-1 is not a reliable indicator of thermogenesis, though it may be a consequence of UCP-1 activation (Ricquier et al., 2000). Increased binding of GDP to mitochondria isolated from brown adipose tissue is a better indicator of UCP-1 activation (Trayhurn and Milner, 1989) activation; this technique also seems to work for skeletal muscle mitochondria (Yoshida et al., 1998).

### Detection of rapid responses to genetic modification

Detection of the acute effects of genetic manipulations on energy expenditure presents a significant challenge. It may be possible to induce genetic manipulation sufficiently rapidly, using for example a tetracycline-inducible site-specific recombinase system such as Cre-*loxP*, for the effect on energy expenditure to be assessed before there is a significant change in body composition (Zhang et al., 2012a). Energy expenditure does not appear to have been measured in any such animal, however. Another possible approach involves the use of adenoviral vectors. These have been used to express a protein that is cleaved to the myokine irisin in skeletal muscle, and irisin was claimed to increase energy expenditure (Bostrom et al., 2012). However, it was unclear how rapidly this effect developed, or whether energy expenditure was expressed per animal or relative to body weight. In another study, adeno-associated viral vectors were used to delete the hypothalamic CB1-R. Unfortunately energy expenditure was not measured until long after the body weights of the control and treated mice had diverged (Cardinal et al., 2012). Therefore, the methods used to assess whether genetic modification of a potential drug target affects energy expenditure are limited to non-acute thermogenesis, as described below.

## Detection of non-acute thermogenesis

### Correction for differences in body composition

It should be more difficult to argue that a compound stimulates thermogenesis if its effect is not immediate or at least fails to appear within a day or two. After this time, energy expenditure might be affected by altered body weight or body composition. Compounds are more likely to affect lean body mass if animals are young (Rothwell and Stock, 1988; Arch et al., 1991) but few studies are conducted in older rodents. If a drug reduces body weight, especially lean body mass, this may mask its thermogenic effect. Studies in genetically modified animals present the same problems. Nevertheless, it is almost routine to read that lean animals have a higher energy expenditure than their more obese counterparts. The device used to justify such claims is to express energy expenditure relative to body weight or body weight^0.75^. In the great majority of cases, the researchers then find that the leaner animals have the higher, mass-specific energy expenditure (Tschop et al., 2012). Those who study energy expenditure in humans have long-recognized that to understand the role of energy expenditure in the aetiology of obesity requires a more sophisticated approach than this. If rodent data are to be treated differently from human data there needs to be a justification, but no such justification has been provided.

#### Prediction of energy expenditure in humans

The illogicality of dividing energy expenditure in rodents by body weight or body weight^0.75^ in order to compare obese with lean rodents and why this can lead to erroneous conclusions has been discussed on a number of occasions (Arch et al., 2006; Butler and Kozak, 2010; Kaiyala et al., 2010; Cannon and Nedergaard, 2011; Even and Nadkarni, 2012; Tschop et al., 2012), including an article in this issue (Speakman, 2013). We shall not reproduce all these arguments but wish to demonstrate its absurdity by applying it to data for humans.

Equation 1 shows the correlation between 24-h energy expenditure (24EE; kcal) and body weight (BW in kg) in 177 male and female subjects with percentage body fat ranging from 3 to 50% (Ravussin et al., 1986).

(1) 24EE=1043+13.0BW

The equation predicts that subjects that weigh 70 kg and 100 kg (which equate to body mass indices of 22.8 and 32.7 for a height of 1.75 m) will have 24-h energy expenditures of 1953 and 2343 kcal respectively—a 20% higher value in the heavier (and obese) subject. If 24-h EE is divided by body weight, however, as is common practice for rodent energy expenditure, we get 27.90 and 23.43 kcal/kg for the 70 and 100 kg subjects respectively—a 16% *lower* value in the heavier and more obese subject.

Similar predictions result from equations for REE, thereby excluding the possibility that differences in energy expenditure between lean and obese subjects are due to differences in locomotor activity.

(2) REE(kcal/d)=879+10.2BW

For example, Equation 2 (Owen et al., 1987) predicts REE values of 1593 and 1899 kcal/d for 70 and 100 kg men respectively—a 19% higher value in the heavier subject. By contrast, the values are 22.8 kcal/kg/d and 18.99 kcal/kg/d for the 70 and 100 kg men respectively—a 17% *lower* value in the heavier subject. If REE is expressed relative to body weight^0.75^, the value is 9% lower in the heavier subject.

There is some evidence that obese subjects have a lower energy expenditure than lean subjects, but no workers have claimed such marked differences. Moreover, they have not used the simplistic approach of dividing energy expenditure by body weight but have adjusted their data for differences in body composition (Ravussin et al., 1988; Major et al., 2007). The reason why energy expenditure per kg body weight is lower in obese subjects is that fat mass has less influence than fat-free mass (FFM) on energy expenditure. Note that any influence that fat mass has on energy expenditure is little to do with the energy required for triglyceride turnover or even the energy requirements of white adipose tissue in which the triglyceride is stored, but may be mainly a consequence of the effects of adipokines, notably leptin, on energy utilization in other tissues (Even et al., 2001; Kaiyala et al., 2010; Wang et al., 2010b; Kaiyala and Schwartz, 2011).

Equations that relate either total or REE to body composition (FFM and %fat) demonstrate the greater influence of FFM compared to fat mass on energy expenditure:

(3) 24EE=488+25.8FFM+4.8% fat

(4) REE(kcal/d)=560+5.39BW+14.14FFM

Equation 3 (Ravussin et al., 1986) predicts that a subject weighing 70 kg of which 10 kg is fat will have a 24-h energy expenditure of 2104 kg, whereas a subject weighing 100 kg in whom all the extra weight is fat will have a 24-h energy expenditure of 2228 kcal—a difference of 124 kcal. On the other hand a muscular 100 kg subject in which the extra 30 kg is FFM (in total 90 kg FFM; 10 kg fat) will have a 24-h energy expenditure of 2858 kcal—a difference of 754 kcal from the 70 kg subject. Thus, extra lean tissue has 754/124 = 6.1 times the effect of extra fat on 24-h energy expenditure.

Equation 4 (Horie et al., 2011) predicts differences of 162 and 585 kcal depending on whether the extra tissue in the 100 kg person is due to extra fat or FFM—a 3.6-fold greater contribution from FFM.

The ratio of 6:1 for the relative contributions of equal weights of FFM and fat to 24-h energy expenditure is within the range of 5.0–6.7 calculated from experiments in mice for REE (Speakman and Johnson, 2000; Selman et al., 2001). Others have reported a ratio as low as 2 for total energy expenditure, which is below the ratio of 3.6 predicted by Equation 4 for REE in humans. The *Lep*^°*b*^/*Lep*^°*b*^ mouse was an exception: fat mass was not a significant independent determinant of energy expenditure unless *Lep*^°*b*^/*Lep*^°*b*^ mice were injected with leptin, its effect apparently being to increase the metabolic energy cost of lean body mass (Kaiyala et al., 2010; Kaiyala and Schwartz, 2011).

#### Analysis of rodent data

Speakman and other authors have argued that regression models should be used to compare energy expenditure data between lean and obese rodents (Arch et al., 2006; Kaiyala and Schwartz, 2011; Tschop et al., 2012; Speakman, 2013). The simplest regression method is to use analysis of covariance (ANCOVA) to relate energy expenditure to body weight for the whole data set—both control and treated or genetically altered groups. Energy expenditure values are shifted in parallel with the slope of the regression line to the mean body weight for the whole data set and then compared between groups (Speakman, 2013). More sophisticated methods involve multiple regression, for example separating out fat mass and lean body mass. It may be even better to separate out components of lean body mass, especially skeletal muscle mass, because this makes a relatively low contribution per unit mass to resting metabolic rate (Even et al., 2001). However, the more components that are included in the analysis, the lower its power to detect differences (Even and Nadkarni, 2012).

Regression analysis is undoubtedly more rigorous than division of energy expenditure by body weight, body weight^0.75^ or even lean body mass. It shows whether differences in energy expenditure might be explained by the effect of a treatment or genetic manipulation on body size or composition (“repartitioning,” which is discussed further below). It may not always be possible to obtain information on body composition, however, especially components of lean body mass, during the in-life phase of a study. Other problems for some data sets are discussed in detail by Speakman (2013). One is that weights used for the regression analysis may not overlap sufficiently between treatment groups or genotypes. Consequently, the regression line may simply join the mean energy expenditure values for the two data sets, so that at each end there are equal numbers of points above the regression line. This inevitably leads to the conclusion that the data set belong to the same regression line. A wider range of weights might give a steeper slope with energy expenditure values for animals from the leaner group tending to lie above the line and those for the fatter group below the line. This problem is more likely to occur with small data sets.

A possible solution might be to fix the slope of the regression line using information for a larger number of animals of the same control and test types, rather than using data from the smaller experiment being analyzed. This would require that the slopes are similar for animals of the types being compared. Biologists take such an approach routinely when they use parametric statistics to compare data sets where *n* < 5, because it is only when *n* ≥ 5 that it can be shown with reasonable power using Bartlett's test whether data have similar variances (exhibit homoscedasticity). Fortunately, we are allowed to use parametric statistics if previous work shows that similar data sets have similar variances (possible after transformation). Without this rule, we could never say that the data sets 1, 2, 3, and 1001, 1002, 1003 are significantly different because the non-parametric Mann–Whitney two-sided *U*-test gives *P* = 0.1.

Even if body weights or the weights of body components overlap, small data sets increase the probability that the regression line will not be significant, especially if weights of individuals are similar in each group. It has been argued that in such cases it is acceptable to divide energy expenditure by lean body mass because it contributes more than fat mass to whole body energy expenditure (Butler and Kozak, 2010). Fat mass cannot be ignored, however, and Speakman (2013) argues that dividing energy expenditure by lean body mass does not increase the power to separate data sets.

This raises the point that the great majority of studies on the effect of genetics on energy expenditure have been underpowered (Tschop et al., 2012; Speakman, 2013). Some workers have gone so far as to suggest that indirect calorimetry is not sufficiently accurate to be of value in long term studies of energy balance (Even and Nadkarni, 2012). Whilst true in many cases, there may be a few mechanisms, such as those that impact on sympathetic activity or the response of brown adipose tissue to sympathetic activity, that have sufficiently large effects for differences in energy expenditure to be detected. For example, in our hands the *Lep*^°*b*^/*Lep*^°*b*^ mouse had a lower energy expenditure per animal than wild-type mice, but only at a temperature below thermoneutrality (Trayhurn and James, 1978; Wilson et al., 1984). In addition, pair-feeding studies in which young *Lep*^°*b*^/*Lep*^°*b*^ mice were yoked to the *ad libitum* energy intake of their lean siblings became obese, reflecting a lower level of energy expenditure, and these differences were much less marked at thermoneutrality than at lower environmental temperatures (Thurlby and Trayhurn, 1979). This phenotype may be due to the *Lep*^°*b*^/*Lep*^°*b*^ mouse not raising sympathetic activity adequately in response to cold (Reichling et al., 1988). [Others have found that *Lep*^°*b*^/*Lep*^°*b*^ mice have a higher energy expenditure than their wild type controls possibly because they measured energy expenditure under different conditions (Himms-Hagen, 1997; Butler and Kozak, 2010)].

The *Lep*^°*b*^/*Lep*^°*b*^ mouse is of course an example of an obese mutant, whereas the focus of this article is leanness. It is therefore disappointing that energy expenditure has not been measured in “skinny” mice, which overexpress leptin, although increased insulin sensitivity has been described (Ogawa et al., 1999). Mice in which the soluble leptin receptor was overexpressed had increased energy expenditure relative to lean body mass, but they also had lower amounts of both lean and fat mass, making interpretation of this finding difficult for reasons explained above (Lou et al., 2010).

In those cases where the data are not suitable for analysis using regression models, the best solution may be to adjust energy expenditure data according to the size of key organs using factors described in the literature (Elia, 1992; Even et al., 2001; Wang et al., 2010a,b). An interesting suggestion is that energy expenditure for mice of differing adiposities might be divided by lean body mass plus 0.2 × fat mass (Even and Nadkarni, 2012). This is consistent with one of the reports discussed above (Selman et al., 2001), which suggested that, relative to its weight lean, body mass contributes five times more than fat mass to energy expenditure. This approach is not ideal, however, and must not be used thoughtlessly, especially in animals that lack a functional leptin system (Kaiyala et al., 2010) As mentioned in section Prediction of Energy Expenditure in Humans, the influence that adipose tissue has on energy expenditure may be mainly a consequence of the effects of adipokines on energy expenditure in other tissues, so the consequences of manipulating adipokine secretion must be considered.

### Non-acute stimulation of energy expenditure per animal

#### Repartitioning

There is little difficulty in arguing that a compound is thermogenic, or a genetically modified mouse suggests a target for thermogenic drugs if energy expenditure *per animal* is increased, whilst body fat is decreased (or at least unchanged) and lean tissue is not increased. A compound that increases energy expenditure per animal because it increases the proportion of more metabolically active organs, such as liver or brain, relative to skeletal muscle might be better described as a repartitioning or anabolic agent, rather than a thermogenic drug. A possible example from our own work is the 11β-hydroxysteroid dehydrogenase type 1 inhibitor BVT.2733, which increased both lean body mass and energy expenditure (Wang et al., 2006). The comparison in this example was with pair-fed mice because BVT.2733 reduced food intake and so its effect was to prevent the loss of lean tissue in response to reduced food intake.

Genetic modifications that increase energy expenditure per animal because they alter body composition may suggest targets for repartitioning agents. Thus, in our own work, both lean body mass and energy expenditure were higher in male acyl CoA:diacylglycerol acyltransferase 1-null than in wild-type mice when the mice were fed on a high fat diet (Wang et al., 2007). Some compounds or genetic modifications may increase energy expenditure due to both a repartitioning effect and a thermogenic effect that is independent of the repartitioning effect. For example, the β_2_-adrenoceptor agonist clenbuterol may cause thermogenesis in obese (*fa/fa*) rats by stimulating brown adipose tissue and by repartitioning energy to skeletal muscle (Rothwell and Stock, 1987).

#### Non-acute thermogenesis in response to compounds

β_3_-Adrenoceptor agonists and FGF21 are examples of treatments that increase energy expenditure per animal when they are given for a week or more, even though body fat content is by this time decreased and body lean content is not increased (Arch and Ainsworth, 1983a; Xu et al., 2009). Leptin may also have increased energy expenditure per animal when given for more than a week to rat pups, though data were expressed relative to body weight, which may have decreased slightly (Stehling et al., 1996). In adult mice, leptin had a more subtle effect that developed over days: it reduced the daily minima of energy expenditure when the mice were food restricted (Doring et al., 1998). Chemical sympathectomy reduced weight loss in response to intracerebroventricular infusion of leptin by 60% over 10 days in rats, despite it not affecting the response of food intake to leptin (Dobbins et al., 2003). This suggests that weight loss was partly due to thermogenesis driven by the sympathetic nervous system. It is consistent with the thermogenic effect of leptin on days 2–6 being reduced by about 50% in mice that lack all three β-adrenoceptors (see section Acute Stimulation of Energy Expenditure). However, the same paper reports that leptin did not cause weight loss over 6 days in either wild type or “β-less” mice other than by reducing food intake (Asensio et al., 2008).

The examples discussed so far are all treatments that also increase energy expenditure almost immediately (β_3_-adrenoceptor agonists) or at least within 2 days (FGF21; leptin). It is much more difficult to find examples of compounds that increase energy expenditure per animal after more than 2 days but not earlier (without increasing lean body mass). B- and C-type natriuretic peptides (BNP and CNP), which activate receptors that are linked to guanylyl cyclases, seem to promote thermogenesis in brown adipose tissue. BNP increased oxygen consumption per mouse after it had been infused for 7 days but its acute effect was not reported (Inuzuka et al., 2010; Bordicchia et al., 2012).

Most claims of increased energy expenditure in lean animals are shown to be ill-founded on detailed examination. For example, antagonism of the activin receptor IIB by immunological means has been claimed to increase energy expenditure either by increasing muscle mass or by increasing brown adipocyte adipogenesis (Fournier et al., 2012; Koncarevic et al., 2012). Energy expenditure was measured after 28 or 60 days, however, and expressed relative to body weight, which in at least one of these studies (Koncarevic et al., 2012) was decreased in proportion to the increase in energy expenditure per gram body weight. Another recent example is JD5037, which is a CB1-R inverse agonist that has poor brain penetration. JD5037 reduced food intake and body weight in diet-induced obese mice (Tam et al., 2012). It was claimed that it increased energy expenditure, but this was expressed relative to body weight^0.75^ and it can be calculated that the ratio of the oxygen consumption values in treated and control mice on day 21 of treatment is very similar to the inverse of the ratio of the body weight^0.75^ values at that time. In other words, energy expenditure per mouse was not altered by treatment. JD5037 affects body weight by reversing leptin resistance and leptin increases sympathetic activity, so it would not have been surprising if it had increased energy expenditure per mouse. However, JD5037 reverses leptin resistance by reversing hyperleptinaemia, which would tend to lower sympathetic activity. Thus, the lower concentration of leptin and the increased sensitivity to leptin may have had roughly equal and opposing effects on energy expenditure. [It is worth noting that leptin expression and release is inhibited by the sympathetic nervous system, so leptin production as well as responsiveness to leptin is subject to feedback control (Mantzoros et al., 1996; Trayhurn et al., 1996)].

The AMPK activator AICAR increased energy expenditure per animal after 4 and 8 weeks' administration to rats compared to pair-fed control (Gaidhu et al., 2011). Energy expenditure was measured weekly and there is no evidence given in this paper or elsewhere of it having an earlier or acute thermogenic effect (although an acute lowering of RER was described in section Acute Stimulation of Energy Expenditure). Thus, AICAR may be an example of a treatment whose thermogenic effect takes more than a week to develop. AICAR also reduced fat pad weights compared to the pair-fed controls, which suggests that weight loss may have been due to thermogenesis; but if so it is surprising that fat mass increased at the same rate as in controls between 4 and 8 weeks of treatment even though thermogenesis did not diminish. The effect of AICAR was not that expected for a peripherally acting AMPK activator because energy expenditure increased only during the dark period and was associated with increased locomotor activity. It is well-established that activation of AMPK in the hypothalamus increases food intake, which raises the possibility that increased locomotor activity elicited by AICAR may have been due to enhancement of food seeking behavior. In the light of this discussion the conclusions of a recent review are of interest. These were that AMPK is “always activated by mitochondrial uncoupling” mediated by UCP-1, and may augment the effect of uncoupling but “activation of AMPK alone does not lead directly to an induction of energy expenditure (Klaus et al., 2012).”

#### Non-acute thermogenesis in response to genetic modifications

The UCP-1 knockout mouse is an example of a genetic modification that elicits (paradoxically) a detectable increase in energy expenditure per animal. This difference could not be detected until the ambient temperature had fallen to about 10–12°C, however. It seemed to be due to the mouse being forced to use a mechanism independent of UCP-1 to generate heat. This mechanism must be less efficient than UCP-1 activation in maintaining body temperature (Ukropec et al., 2006a). Another example is the melanin-concentrating hormone 1 receptor knockout mouse, but in this case the increase was associated with increased locomotor activity (Marsh et al., 2002). The renin knockout mouse displayed an increased energy expenditure relative to its lean body mass, and at least in the case of REE, it seems likely that energy expenditure per mouse was raised (Takahashi et al., 2007).

These are rare examples. An example that does not stand up to scrutiny is that overexpression of FGF19 increases energy expenditure (Tomlinson et al., 2002). It was only energy expenditure expressed relative to body weight^0.75^ that was increased. Once again it can be calculated from the data presented that energy expenditure per animal was no different between transgenic and wild-type mice. The authors also argued that energy expenditure must have been increased because food intake was higher in the transgenic mice, but food intake was expressed relative to body weight (not like energy expenditure as body weight^0.75^) and it was not increased on a whole animal basis.

We have already argued that exogenous FGF21 has a thermogenic effect. This was detectable after 19 days of treatment (Xu et al., 2009). It is perhaps therefore surprising that energy expenditure expressed relative to body weight was similar in mice in which FGF21 was overexpressed and wild-type mice, even though the FGF21 overexpressing mice were much smaller. Thus, energy expenditure per animal was lower in the transgenic mice in proportion to their weight. However, the body composition of the transgenic and wild-type mice was similar, so this is not a comparison between a lean and an obese strain. It appears instead to be a situation where energy expenditure really does reflect body size (Zhang et al., 2012b).

There are a few examples of where the correlation between energy expenditure and body weight has been shown to be altered by genetic modification. Some of these involve modifications that affect the development and function of brown adipose tissue. Thus, ANCOVA showed that energy expenditure at a given body weight was increased in mice with a null mutation for transient receptor potential vanilloid 4 (TRPV4). An antagonist of TRPV4 increased the expression of UCP-1 in brown adipose tissue, but its effect on energy expenditure was not reported (Ye et al., 2012). Mice in which UCP-1 was overexpressed in skeletal muscle were leaner than wild-type mice (unlike the UCP-1 knockout mice, this is predictable rather than paradoxical). Mean energy expenditure was not increased, but for a given body weight it was higher in the genetically modified animals (Couplan et al., 2002). It might be argued that this was because the weights of the brain and liver—organs that make a disproportionate contribution to energy expenditure—were increased. However, other work showed that energy expenditure (which was expressed relative to body weight) was increased at night but not during the day, despite there being no difference in locomotor activity between the wild-type and transgenic mice. Daytime energy expenditure was, therefore in effect, a within-animal control for night-time energy expenditure. The authors therefore concluded that muscle energetic efficiency was decreased by over-expression of UCP-1 in skeletal muscle (Klaus et al., 2005). The fact that activation of UCP-1 is the mechanism of thermogenesis in brown adipose tissue seems to support the view that this is a genuine example of a genetic modification that stimulates thermogenesis, but as mentioned previously, the expression of UCP-1 indicates only the capacity for thermogenesis in brown adipose tissue and not whether this capacity is being used. Other examples of the effect of overexpression of UCP-1 in skeletal muscle, white adipose tissue and liver have been reviewed recently in relation to the role of AMPK in mediating the phenotype (Klaus et al., 2012).

### Indirect evidence

It may be possible to deduce that a compound or genetic modification has increased energy expenditure without directly measuring expenditure itself. One such situation is where energy intake is increased and yet the treated or genetically modified animal has a reduced body fat content and no increase in lean body mass. For example, repeated administration of the β_3_-adrenoceptor agonist BRL26830 increased food intake in lean mice but reduced their body lipid content and had no effect on lean body mass (Arch and Ainsworth, 1983b). In this instance it was also apparent from indirect calorimetry that energy expenditure per animal was elevated. Similarly, 11β-hydroxysteroid dehydrogenase type 1 knockout mice fed on a high fat diet were less obese than wild-type mice, despite having a higher caloric intake (Morton et al., 2004). Their increased core temperature also suggested that energy expenditure was raised. By contrast, increased energy expenditure per animal was not evident in mice that lacked acetyl-CoA carboxylase-2, but it appeared that energy expenditure must have been increased because the knockout mice consumed more energy than the wild-type mice but had a reduced body fat and lean content (Abu-Elheiga et al., 2001; Choi et al., 2007). The knockout mice showed increased energy expenditure relative to lean body mass, but it can be calculated from the data provided that energy expenditure per animal was no different between the knockout and control mice (Choi et al., 2007). It is difficult to understand how energy intake per mouse could have been so much higher (20–30%) in the knockout than the wild-type mice and yet energy expenditure per mouse was not increased. Other workers have failed to replicate these findings (Hoehn et al., 2010; Olson et al., 2010). Nevertheless, the principle that it may be possible to deduce from energy intake and body composition that energy expenditure has increased remains valid. This assumes, of course, that the food eaten is absorbed normally and energy is not lost as a result of glycosuria or ketosis; in other words, that the metabolisability of the diet is not altered.

Another situation that might suggest increased thermogenesis is where body fat content is reduced, but there is no decrease in food intake, or food intake is kept equal between groups by pair-feeding. The problem with this argument is that it is easier to show that body fat content differs between two groups of animals than that their energy intake (especially energy absorption) differs. Without positive evidence of increased energy expenditure such arguments are usually unreliable. A similar argument is that drugs that are both acutely thermogenic and acutely anorectic cause fat loss in the longer term through thermogenesis, because the effect on food intake usually wanes rapidly and food intake over the whole course of the study is not reduced (Arch, 1981; Day and Bailey, 1998; Picard et al., 2000; Billes and Cowley, 2008; Herling et al., 2008). This logic is of little value, however, unless it is demonstrated that the thermogenic effect of the treatment does not disappear in the same way that the anorectic effect disappears.

One instance where the thermogenic effect of treatment did not disappear was when the *Lep*^°*b*^/*Lep*^°*b*^ mouse was dosed repeatedly with a β_3_-adrenoceptor agonist (Arch and Ainsworth, 1983a). The *Lep*^°*b*^/*Lep*^°*b*^ mouse is amenable to such a finding because it has low sympathetic activity at temperatures below thermoneutrality and it has a lot of fat to lose. Their lean littermates do not have as much fat in their whole body as the *Lep*^°*b*^/*Lep*^°*b*^ mouse loses in response to treatment with β_3_-adrenoceptor agonists for a month. Consequently, since lean body mass is not reduced, lean mice must either have a reduced thermogenic response as treatment continues or they must increase their energy intake (Arch and Ainsworth, 1983a). Unfortunately, the *Lep*^°*b*^/*Lep*^°*b*^ mouse is not a good general model of human obesity because it lacks leptin, and this has many downstream consequences—low sympathetic activity and high hypothalamic neuropeptide Y release, for example. Thus, any intervention that corrects leptin deficiency or any of its downstream consequences is likely to have an exaggerated effect in the *Lep*^°*b*^/*Lep*^°*b*^ mouse.

Full carcass analysis coupled with careful measurement of metabolisable energy intake is occasionally used to calculate energy expenditure (Dulloo and Miller, 1987; Mercer and Trayhurn, 1987). Ideally, such studies should be conducted using metabolic chambers and faecal and urinary energy should be measured. This approach has been used to demonstrate that the β_2_-adrenoceptor agonist clenbuterol increases thermogenesis as well as increasing lean tissue at the expense of fat (Rothwell and Stock, 1987).

## Translation of rodent findings to humans

It is important to understand how, and when (i.e., under what conditions), a compound increases energy expenditure in rodents before investigating whether it does the same in humans. Similarly, it is sensible to know how a genetic modification increases energy expenditure before seeking drugs that act via the product of the modified gene. An obvious example of the “how” issue is where thermogenesis is associated with increased locomotor activity. Whether this is an acceptable mechanism of action for an antiobesity drug is debatable, but it clearly would make no sense to expect energy expenditure to be raised in humans when they are resting. Thyroid hormone-related mechanisms raise the spectre of cardiac stimulation, loss of skeletal muscle, bone wasting, fatigue, and CNS effects (Crunkhorn and Patti, 2008), although there has been interest in the possibility that selective activators of thyroid hormone receptor-β might avoid such problems (Ribeiro, 2008).

The significance of the “when” question can be illustrated by the influence of ambient temperature on the phenotype of the UCP-1 knockout mouse. The phenotype of these mice appears to depend on whether they are housed at thermoneutrality or below thermoneutrality. Reports vary, however, from the UCP-1 knockout mouse being obese and especially sensitive to diet-induced obesity when housed at thermoneutrality (Feldmann et al., 2009), to their being resistant to diet-induced obesity when housed at 20°C (Liu et al., 2003). Most humans live at temperatures near thermoneutrality, which suggests that, if anything, *activation* of UCP-1 in brown adipose tissue, rather than its absence should increase whole body thermogenesis. However, there is the possibility that less efficient thermogenic mechanisms in other tissues might shut down when brown adipose tissue is activated, entirely negating the effect of UCP-1 activation.

A common situation is that a compound increases energy expenditure because it increases the activity of the sympathetic nervous system. Activation of the sympathetic nervous system in humans has far less effect on energy expenditure in humans than in rodents. The maximum increase in energy expenditure in humans at thermoneutrality in response to sympathomimetic drugs is about 30% (Schiffelers et al., 2000, 2001), whereas in rodents increases of two- or threefold are possible if the animals are maintained at around 21°C (Wilson et al., 1984; Wernstedt et al., 2006; Feldmann et al., 2009). Even greater fold increases are possible if the animals are maintained at thermoneutrality to suppress their baseline sympathetic activity. This also applies to a centrally acting agent such as ephedrine: it should not be assumed that centrally acting drugs cannot raise sympathetic activity if rodents or humans are at thermoneutrality (Wilson et al., 1984; Gordon, 2012). It is important to remember that the thermoneutral zone may be lowered if animals are housed in groups so that they can huddle, or if they are given plenty of bedding to wrap up in or a wheel to keep active; this is reflected in the differences in thermogenic activity and capacity of their brown adipose tissue when mice are housed singly or in groups of varying size (Jennings et al., 1986). Large effects may also occur in *Lep*^°*b*^/*Lep*^°*b*^ mice and similar models of leptin dysfunction, which have low sympathetic activity (Wilson et al., 1984). Indeed, it is possible that increased energy expenditure might not translate at all from such models to humans.

To give some perspective on thermogenesis in humans, a simple calculation shows that anorectic drugs must alter energy balance by about 10% to cause weight loss of about 5 kg over six months, which historically has been a typical effect (Rucker et al., 2007). It may be difficult to achieve this over 24 h if the maximum effect is 30%. On the other hand, despite their relatively low efficacy as stimulants of energy expenditure, sympathomimetic drugs, such as β-adrenoceptor agonists, have been shown to improve insulin sensitivity markedly in humans (Mitchell et al., 1989; Smith et al., 1990).

What if a compound does increase sympathetic activity in humans? Might it have undesirable side-effects due to generalized sympathetic activation? This was the case with sibutramine. It is unclear whether thermogenesis contributed to its antiobesity effect, but sympathetic activity contributed to the cardiovascular side effects that eventually led to its withdrawal (James et al., 2010). The thermogenic effect of rimonabant, by contrast, was not detected in humans, although another CB1-R antagonist did raise energy expenditure (Addy et al., 2008).

A drug that raises sympathetic activity selectively in brown adipose tissue, skeletal muscle or white adipose tissue, rather than in the cardiovascular system would be ideal, but is this feasible? Despite evidence that sympathetic nerves to brown adipose tissue can be activated selectively (Morrison, 2001; Kosari et al., 2011), leptin, the main role of which is to regulate energy balance, raises sympathetic activity in “non-metabolic” tissues (Haynes et al., 1997; Rahmouni and Morgan, 2007). The current interest in drugs and drug targets that increase the response of brown adipose tissue to the sympathetic nervous system offers a possible way to avoid these problems.

## Conclusion

As long as we seek drugs for the treatment of obesity, the questions, “Is it thermogenic?” and “Is it a target for thermogenic drugs?” will continue to be asked. It is a relatively simple matter to show whether a compound stimulates thermogenesis if energy expenditure increases rapidly and cannot be a consequence of altered body composition. The clearest examples of such compounds are those that activate or mimic the sympathetic nervous system, and so it is wise to check whether any compound whose effect appears rapidly acts via such a mechanism.

Even if a compound has an anti-obesity effect and is acutely thermogenic, it cannot be assumed that this is why it causes weight loss unless it raises 24-h energy expenditure throughout the weight loss experiment. This then raises an issue that also afflict studies in which potential drug targets are genetically modified in mice: how should energy expenditure be expressed. The wrong way, at least when comparing lean and obese mice, is to divide energy expenditure by body weight or body weight^0.75^. The surest way is to express energy expenditure per mouse and then to check whether energy expenditure has increased because there is an increase in the absolute amount of lean tissue—in other words the treatment would be better described as “repartitioning” rather than thermogenic.

There are other ways of detecting thermogenesis than measuring energy expenditure, and there are ways of comparing energy expenditure measurements that allow for differences in body weight and composition, but they should not be used without an understanding of their limitations.

### Conflict of interest statement

The authors declare that the research was conducted in the absence of any commercial or financial relationships that could be construed as a potential conflict of interest.
